# The effects of leptin on human cytotrophoblast invasion are gestational age and dose-dependent

**DOI:** 10.3389/fendo.2024.1386309

**Published:** 2024-05-23

**Authors:** Kristen K. Rumer, Shilpi Sehgal, Anita Kramer, Kevin P. Bogart, Virginia D. Winn

**Affiliations:** ^1^ Department of Obstetrics and Gynecology, University of Colorado, Aurora, CO, United States; ^2^ Department of Molecular and Cellular Medicine, University of Colorado, Aurora, CO, United States; ^3^ Department of Obstetrics and Gynecology, School of Medicine, Stanford University, Stanford, CA, United States

**Keywords:** invasion, leptin, leptin receptor, placenta, preeclampsia, trophoblast

## Abstract

**Introduction:**

Leptin and its receptors are expressed by the human placenta throughout gestation, yet the role of leptin in early human placental development is not well characterized. Leptin is overexpressed in the placentas from preeclamptic (PE) pregnancies. PE can result from the impaired invasion of fetal placental cells, cytotrophoblasts (CTBs), into the maternal decidua. We hypothesized that elevated leptin levels would impair human CTB invasion.

**Methods:**

The effects of leptin on the invasion of human CTBs were evaluated in three cell models, HTR-8/SVneo cells, primary CTBs, and placental villous explants using invasion assays. Further, leptin receptor expression was characterized in all three cell models using RT-PCR. Further phosphokinase assays were performed in HTR-8/SVneo cells to determine signaling pathways involved in CTB invasion in response to differential leptin doses.

**Results:**

We found that, prior to 8 weeks gestation, leptin promoted CTB invasion in the explant model. After 11 weeks gestation in explants, primary CTBs and in HTR-8/SVneo cells, leptin promoted invasion at moderate but not at high concentrations. Further, leptin receptor characterization revealed that leptin receptor expression did not vary over gestation, however, STAT, PI3K and MAPK pathways showed different signaling in response to varied leptin doses.

**Discussion:**

These data suggest that the excess placental leptin observed in PE may cause impaired CTB invasion as a second-trimester defect. Leptin’s differential effect on trophoblast invasion may explain the role of hyperleptinemia in preeclampsia pathogenesis.

## 1 Introduction

Leptin, a 16kDa multifunctional peptide hormone secreted primarily by adipocytes, is also secreted by the placenta (1, 2). Leptin’s role has been implicated in energy homeostasis, immune function, and reproduction (3–5). Throughout human gestation, placental trophoblasts produce leptin and express leptin receptors (LepR) suggesting leptin likely plays a role in placental development (6, 7). Leptin levels in pregnancy depend both on maternal body mass index (BMI) and gestational age (8, 9). Over the course of gestation, as the placenta enlarges, circulating leptin levels increase, peak in the second trimester, and fall back to normal levels postpartum (10).

There are several lines of evidence to indicate that leptin is critical in promoting the invasion of placental cytotrophoblasts (CTB). In the homozygous female ob/ob (leptin-deficient) mouse, early placental invasion can only occur if the mice are administered with exogenous recombinant human ob protein, leptin (11, 12). Further, leptin promotes invasion of primary mouse CTB in a matrix metalloproteinase (MMP) dependent manner. The promotion of murine CTB invasion is seen only in early gestation and is lost in later gestation (13). Leptin has been demonstrated to exert a dose-dependent effect on MMPs and fetal fibronectin in human cytotrophoblast cells implicating its role in CTB invasion (14). However, the dose-dependent effect of leptin on human pCTB invasion has not been directly determined.

Several key features of a human pregnancy suggest that leptin may have different effects on CTB invasion compared to what has been demonstrated in mice. Mammalian placental structure and development are not conserved. Humans have one of the most invasive placentas; human CTBs invade through the decidua into the myometrium to establish the basal plate. By contrast, murine CTBs invade only to a shallow depth in the decidua (15). Further, primarily only human pregnancies are affected by preeclampsia (PE), a disorder characterized by maternal hypertension and proteinuria or other end organ damage after 20 weeks of gestation, which can arise from inadequate CTB invasion earlier in pregnancy (16). PE has not been observed to occur spontaneously in other species except higher apes (17). Increased leptin at the protein and/or transcript level has been reported in the maternal serum and placentas of preeclamptic women as compared to controls even after accounting for maternal BMI (18–30). As early as 13 weeks of pregnancy, a woman’s risk of PE increases with increasing serum leptin levels. In fact, for every 10ng/ml increase in serum leptin, the risk of preeclampsia increases by 30% (31, 32). Interestingly, one of the risk factors for PE is obesity which has the characteristic of hyperleptinemia (33). These associations between leptin and PE raise the question of whether leptin impairs human CTB invasion. We hypothesize that high levels of leptin inhibit human CTB invasion. To test this hypothesis, the effects of varying concentrations of leptin on human trophoblast invasion of early and mid-gestation human CTBs were evaluated using HTR-8/SVneo cells, primary CTBs, and placental villous explants. Further, the expression of LepR was characterized and the leptin impact on downstream signaling pathways was explored. Understanding the effects of leptin on human CTB invasion is important for a better understanding of both normal placentation and PE pathogenesis.

## 2 Methods

### 2.1 Tissue collection

Biopsy specimens of tissue from elective pregnancy terminations were collected using protocols approved by the University of Colorado Institutional Review Board. Exclusion criteria included evidence of infection, hydropic changes, and known genetic or fetal anomalies. Gestational age was determined using standard dating criteria (34, 35). Tissue was washed extensively in cold phosphate-buffered saline (PBS, RICCA Chemical Company) and processed within one hour of the procedure. Tissue was immediately processed for isolation of primary CTBs (pCTB), villous explants, formalin-fixed for histology, or snap frozen and stored at -80°C for later RNA or protein isolation.

### 2.2 HTR-8/SVneo cell culture

HTR-8/SVneo immortalized first-trimester human trophoblast cells (derived by Charles Graham (36) were cultured in RPMI 1640 medium (Hyclone) supplemented with 5% fetal bovine serum (Hyclone), 100 U/ml penicillin and 100 g/ml streptomycin (Invitrogen). Cells were maintained in monolayer cultures on plastic at 37°C in 5% CO_2_, 95% air, and 95% humidity.

### 2.3 Primary cytotrophoblast and villous explant isolation

To confirm findings obtained with the HTR-8/SVneo cells with more physiologic systems we used both primary CTB and villous explants. Primary CTB isolations were done as previously described (37). Briefly, villi were dissected away from the chorionic and basal plates to isolate the pure population of primary CTBs. A series of enzymatic digestions were used to isolate CTBs which were further purified over a Percoll gradient. The purity of each CTB preparation was determined by spinning aliquots of each preparation onto slides (Shandon Cytospin 4, Thermo Scientific) for immunofluorescent staining. Cells were fixed with 4% paraformaldehyde for one hour, permeabilized with 10% cold methanol for 10 minutes, and blocked by incubating with 3% BSA (wt/vol), 0.1% Triton X-100 (vol/vol), and 0.5% Tween 20 (vol/vol) in PBS for one hour at room temperature. The cells were incubated overnight at 4°C with anti-cytokeratin-7 antibody (1:100, Dako, clone OV-TL 12/30) to identify trophoblasts, anti-vimentin antibody (1:200, Abcam, clone SP20) to identify contaminating stroma or decidual cells or anti-CD45 antibody (1:50, Dako, clone 2B11+PD7/26) to identify contaminating immune cells. Species-appropriate Alexa Fluor conjugated antibodies (1:1000, Invitrogen) were used as secondary antibodies. Nuclei were stained with DAPI and the cells were mounted in Vectashield containing 4’,6-diamidino- 2-phenylindole (Vector Laboratories). Imaging was done using a Leica DM 5000B fluorescence microscope equipped with a Leica DFC 350FX digital camera (Leica Microsystems, Bannockburn, IL; and Leica-Camera, Solms, Germany). Cytotrophoblast purity ranged from 80-95+% and the degree of purity did not affect the observed phenotype.

Villous explants were isolated as previously described (38, 39). Villous tissue (~5mm) containing 1-4 cell columns was collected from 5-11 weeks gestation and was plated on a 24-well hanging chambers with 0.4 μm pores (Millipore) coated with 120 µl of Matrigel (BD Bioscience) per well. The explants were incubated overnight in 20 µl explant medium (DMEM: F12K medium containing 10% fetal bovine serum, 100 U/ml penicillin, and 100 µg/ml streptomycin) in the top of the chamber and 500 µl explant media in the bottom chamber. The following morning, 200 µl of explant medium was added to each chamber. Explants were cultured for an additional 24-48 hours until cell column development was evident; villi lacking cell column development were discarded. Conditioned medium from the top of the wells was then removed and combined. Unused conditioned medium was stored at -20°C for use on subsequent experimental days.

### 2.4 Cell invasion assay

HTR-8/SVneo or pCTBs were seeded in invasion chambers with 8 µm pores size membrane, (Costar) coated with 10 µl of Matrigel Basement Membrane Matrix. (BD Bioscience) (20,000 HTR-8/SVneo cells or 200,000-250,000 pCTBs per well). The invasion chambers were placed in a 24-well cell culture plate. Invasion medium [HTR-8/SVneo: RPMI 1640. pCTBs: DMEM high glucose (Hyclone) supplemented with 2% Nutridoma-SP (Roche) and 50 µg/ml gentamycin (Gibco)] containing 0, 10, 80, 160, or 320 ng/ml human recombinant leptin (Sigma) was added in both the upper chamber and the bottom well. The medium was replaced every 24 hours and the invasion cultures were harvested at 48 hours (HTR-8/SVneo) or 72 hours (pCTBs). Chambers were washed three times in PBS, fixed for 20 minutes in 3% paraformaldehyde at 4°C and permeabilized in ice-cold methanol for 5 minutes at 4°C. Invasion chambers were stored at -20°C until immunofluorescent staining. HTR-8/SVneo chambers were rehydrated in PBS and then mounted in a DAPI-containing mounting medium (Vector Labs) to stain the nuclei. The pCTB chambers were rehydrated in PBS and then incubated overnight at 4°C in anti-cytokeratin-7 antibody followed by anti-mouse Alexa Flour 488 antibody for one hour. Invasion was quantified by counting invasive nuclei (HTR-8/SVneo) or cytokeratin-7 positive cellular projections (pCTBs) on the lower surface of the filter membrane. Counts were averaged from 2-5 membranes per leptin concentration per experiment. HTR-8/SVneo experiments were performed three times. Primary CTB isolations from 6 placentas (13–24-week gestations) were evaluated independently. The average invasion at each leptin concentration was normalized to the average invasion at 0 ng/ml leptin. The data were then combined after normalization.

Villous explants were grouped by size and number of cell columns and then randomly assigned to treatment condition; the first treatment day was defined as Day 0. Explants were fed with 70% explant medium, 30% conditioned medium (described above), and 0, 10, 80, 160, or 320 ng/ml leptin in the upper chambers and explant medium with corresponding leptin concentration in the lower chambers. The leptin-containing medium was replenished on Days 1 and 2. Explants were photographed on Days 0, 1, 2, and 3. An Olympus SZX12 dissecting scope equipped with an Olympus DP72 digital camera was used to photograph the explants and the areas of trophoblast outgrowth were manually outlined. The traced area reflects both CTB migration over the surface and invasion into the Matrigel. The area on Day 3 was normalized to the area on Day 0 to account for baseline differences.

### 2.5 Absolute cell number determination

HTR-8/SVneo cells (20,000 cells per well) were plated on 96 well plates coated with 10 µl of Matrigel. Cells were incubated in RPMI 1640 medium containing leptin at 0, 10, 80, 160, or 320 ng/ml. The medium was replaced at 24 hours and the cells were retrieved at 48 hours by trypsinization with mechanical disruption. Cell numbers were quantified using a hemocytometer. Two counts per well with three wells per concentration were averaged at each leptin concentration. The absolute cell number was normalized to the average cell number at plating. The experiment was repeated twice.

### 2.6 Leptin ELISA

Secretion of leptin into the conditioned medium was quantified using an Alpco ELISA for human leptin following manufacturer standard protocol and provided standards. Twenty microliters of conditioned medium samples from invasion assays were analyzed in duplicate. Additional standard curves using the same leptin as used in the invasion assays diluted in appropriate culture medium were also evaluated. The assay had a limit of detection of 0.5 ng/ml, intra-assay variation of approximately 4.6%, and inter-assay variation of 6.1%, as reported by the manufacturer.

### 2.7 Zymography of MMP-2 and -9 activity

Conditioned medium from the HTR-8/SVneo and pCTB invasion assays were collected at 24, 48, and 72 (pCTBs only) hours, frozen in liquid nitrogen, and stored at -80°C. An equal volume of conditioned medium from each experimental condition was treated with Lammeli sample buffer without boiling or reduction. Samples were frozen/thawed in liquid nitrogen/37°C three times before separation on 8% polyacrylamide gels containing 3 mg/ml porcine gelatin A (Sigma) at 100 V for 90 minutes at 4°C. After electrophoresis, the gels were soaked in 2.5% Triton X-100 twice for 30 minutes at RT and incubated in Zymogen buffer (50 mM Tris-HCl pH 8.0 and 5 mM CaCl_2_) at 37°C for 48-72 hours to allow proteinase digestion of its substrate. Gels were stained with Coomassie blue and visualized on a Biorad Chemi Doc XRS. Proteolytic activities appeared as white bands of lysis against a dark background of stained gelatin. Explant cultured medium contains a serum that has inherent MMP activity negating the ability to evaluate MMP activity in the explant model system.

### 2.8 Detection of LepR by immunohistochemistry

Formalin-fixed, paraffin-embedded biopsy tissues were serially sectioned. Five-micrometer-thick tissue sections were cut on a microtome and mounted on super frost glass slides. The sections were deparaffinized and rehydrated before immunostaining. The goat ABC kit (Vector) was used for LepR staining. ObR N20 antibody (1:50) was used to detect all LepR isoforms, ObR C20 antibody (1:100) was used to detect the long isoform (Santa Cruz). Visualization was done with diaminobenzidine (DAB, Dako) and hematoxylin QS (Vector Laboratories) was used for the nuclear counter stain. Negative control staining was performed without primary antibodies. A Nikon Eclipse 80i microscope equipped with a Q-imaging Retiga 100R digital camera and NIS-Elements Advanced Research version 2.30 software was used for visualization and photography. Each sample was evaluated for intensity (1-3) and percent-positive-trophoblasts (0: <10%, 1: 10-50%, 2: 50-90%, 3 >90%) and the values were multiplied to calculate the immunoreactivity score.

### 2.9 RT-PCR to detect LepR isoforms

Total RNA was isolated from HTR-8/SVneo cells, snap-frozen explants, or pCTBs using Trizol reagent following the manufacturer’s protocols (Invitrogen). RNA was quantified by measuring the absorption at 260 nm and stored at −80°C. cDNA was generated with ImProm-II reagents (Promega) from 1 ug of RNA according to manufacturer’s protocols. Templates were amplified with Promega PCR 2X master mix according to standard protocols and primers synthesized by Integrated DNA Technologies (sequences in **Table 1**). 18S primers were used as an amplification control. Primer specificity was confirmed by demonstrating uniqueness though NCBI BLAST and detecting a single amplicon band at the predicted size on an ethidium bromide embedded 1.5% agarose gel. Briefly, RT-PCR was performed under the following conditions: denaturation at 95°C for 2 minutes, 35 amplification cycles of: 95°C for 45 seconds, 60°C for 30 seconds, 72°C for 60 seconds followed by a final extension at 72°C for 10 minutes.

**Table 1:** pan Lep R and isoform-specific primer sequences.

	Forward Primer Sequence	Reverse Primer Sequence	Amplicon Size (base pairs)
LepR long (b)	5’- ACA GCA TCA GTG ACA TGT GGT CCT -3’	5’- - CCA AGG GTT GTC TCT GGC TTT CGT -3’	244
LepR short (a)	5’- - GAA GCC CGA AGT TGT GTT TGT GCT -3’	5’- TTT GCA TCA CGT CCC ATT TCC CGT -3’	212
LepR short (c)	5’- - GAT GCT TGA AGG CAG CAT GTT CGT -3’	5’- TTC TTG GGT TCT CAC AGA GGG -3’	202
18s	5’- - ATC CCT GAA AAG TTC CAG CA - 3’	5’- CCC TCT TGG TGA GGT CCA TG -3’	186

### 2.10 RT-qPCR to detect LepR expression changes in HTR8/SVneo cells treated with increasing leptin dose

1X10^5^ HTR8/SVneo cells were seeded in a 12-well plate and treated with 0,10,80,160 and 320 ng/ml of leptin for 24 and 48 hours. Post-treatment the cells were lysed and total RNA was isolated and quantified. cDNA was generated with iScript (Bio-rad) from 1ug of RNA using the manufacturer’s protocol. qPCR was performed with the fluorescent Taqman method using panLepR primers. The thermal cycling conditions were: 50°C for 2 min (AMPerase activation), 95°C for 10 min (Taq activation), 95°C for 15 sec for denaturation, and 60°C for 1 min for annealing and extension. To determine expression levels of LepR isoforms, qPCR based on the Syber Green fluorescence method was implemented using isoform-specific primers (**Table 1**). The thermal cycling conditions were: 95°C for 2 minutes; 40 cycles of 95°C for 45 seconds and 60°C for 30 seconds; and a melt curve analysis using temperatures from 60°C to 95°C. For quantification of gene expression changes, the 2^-ddCt method was used to calculate the fold change relative to the control (0 ng/ml). The gene expression values for LepR at each dose were normalized to 18S. All measurements were conducted in triplicates.

### 2.11 Phospho-kinase assay

The human phosphokinase array kit (R and D systems was used according to the manufacturer’s protocol. Briefly, HTR-8/SVneo cells were cultured on Matrigel for an hour and then stimulated with 0, 80 or 320ng/ml leptin for 30 minutes (24 well plates, 1:1 Matrigel: Serum-free media, 500,000 cells/well). Wells were washed with PBS and lysates were harvested in lysis buffer. The lysate was centrifuged, and the supernatant was incubated with Blots A and B overnight at 4°C (167ul lysate per part per sample) that had been pre-blocked in array buffer 1. Blots A and B are designed to identify different phosphorylations with minimal cross-reactivity. The blots were washed, incubated in the presence of A or B antibody cocktails, washed, incubated in A and B detection antibody cocktails, and washed again. Streptavidin-HRP detection reagent was then added, and blots were washed one last time. Visualization of the immunoreactive spots was done using chemiluminescence (Western Lightning Plus -ECL, Perkin Elmer) and exposure to XR film (Kodak). Film imaging and spot densitometry were performed using Biorad Chemi Doc XRS and QuantityOne software. Each blot contains duplicate spots for analyses of phosphorylation sites, 46 unique phosphorylation sites between the two blots. The average background signal from negative control spots was subtracted from each individual phospho-spot. The average background-corrected signal of duplicate spots from each treatment condition is reported as a fold change from the average background-corrected signal of the equivalent spots from 0 ng/ml treated lysates.

### 2.12 Statistics

One-way ANOVA with Tukey post-test for multiple comparisons was carried out to determine statistically significant differences, p<0.05 was considered significant.

## 3 Results

To determine the effect of leptin on human CTB invasion, we utilized three different model systems from the first and second trimesters of human pregnancy, the timeframe of maximal CTB invasion. The three systems were HTR-8/SVneo immortalized human trophoblast cells, pCTB and placental villous explants. The absolute concentration of leptin in placental tissue is unknown; however maternal serum levels of leptin vary during pregnancy from 40 to over five hundred ng/ml (40) based on many factors including maternal BMI, gestational age, and disease state such as PE. To model this wide range of serum leptin concentrations, we evaluated the effects of 0-320 ng/ml leptin on the invasion of human CTBs.

### 3.1 Leptin promotes invasion of HTR-8/SVneo cells and primary human CTBs in a dose-dependent manner

**Figure 1:**
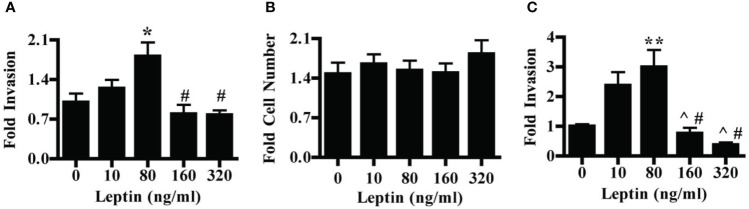
Leptin has a differential effect on HTR-8/SVneo and second-trimester human pCTB invasion. **(A)** Invasion of HTR-8/Svneo cells through Matrigel-coated invasion chambers was evaluated in the presence of 0, 10, 80, 160 or 320 ng/ml leptin. Data normalized to invasion at 0 ng/ml leptin are combined from three independent experiments: n-12, 8, 14, 13, and 12 invasion chambers per respective leptin concentration. **(B)** Absolute cell number of HTR-8/Svneo cells cultured on Matrigel in the presence of 0-320 ng/ml leptin was evaluated. Number of cells after 48 hours was normalized to the number of cells plated at the start of the experiment. Normalized data from three independent experiments were combined, n-12 wells per concentration. **(C)** Invasion of pCTBs plated as described for **(A)** Primary CTBs isolated from six placentas (13-24 weeks gestation) were evaluated independently and normalized data combined; n=20, 19, 19, 13, 7 invasion chambers per respective leptin concentration. Data are reported as mean fold change from 0 ng/ml +/- standard error of mean (SEM). *p<0.05 vs. 0 ng/ml, **p<0.01 vs. 0 ng/ml, ^p<0.05 vs. 10 ng/ml, ^#^p<0.01 vs. 80 ng/ml.

We first evaluated the effect of leptin on the invasion of the HTR-8/SVneo cells, a cell line derived from first-trimester placenta commonly used as an *in vitro* model of non-cancerous human cytotrophoblast and invasive trophoblasts. Leptin had a dose-dependent effect on the invasion of HTR-8/SVneo cells. Treatment of HTR-8/SVneo cells with 80 ng/ml leptin promoted invasion over no leptin treatment. At 160 and 320 ng/ml leptin, the invasion was significantly decreased as compared to invasion at 80 ng/ml but did not differ from invasion in the absence of leptin (**Figure 1A**). To determine whether changes in the invasion were due to actual changes in invasive phenotype and not due to changes in cell number, we evaluated the effect of leptin on HTR-8/SVneo cell number when cultured on Matrigel. HTR-8/SVneo cell number increased over 48 hours in the absence of leptin, but leptin dose did not differentially affect HTR-8/SVneo cell number (**Figure 1B**). Therefore, the differences in the invasion were due to a direct effect of leptin on CTB invasion rather than an alteration in cell number. We next evaluated the effect of leptin on the invasion of primary human CTBs to determine if the dose-dependent effect would be recapitulated in a more physiologic system. Leptin impacted the invasion of second-trimester human pCTBs. As with the HTR-8/SVneo cells, 80 ng/ml leptin promoted maximal invasion. Invasion at both 160 and 320 ng/ml leptin was significantly lower than at 10 and 80 ng/ml leptin but did not differ from invasion in the absence of leptin (**Figure 1C**). This effect is not likely due to alterations in CTB number since pCTBs do not proliferate on Matrigel under these normoxic conditions (41, 42). Of note, no gross differences in apoptotic or mitotic nuclei were observed. As leptin is known to be secreted by the human placenta, we determined if the secretion of endogenous leptin by CTBs in our culture systems could account for the biological differences seen. We measured leptin in the conditioned medium by ELISA. Leptin was not secreted at detectable levels (< 0.5 ng/ml) by HTR-8/SVneo cells or pCTBs during the timeframe of culture on Matrigel. Therefore, endogenous leptin secretion is unlikely to be influencing the observed phenotypes.

### 3.2 Leptin’s impact on the invasion of explant CTBs varied by gestation and dose

**Figure 2:**
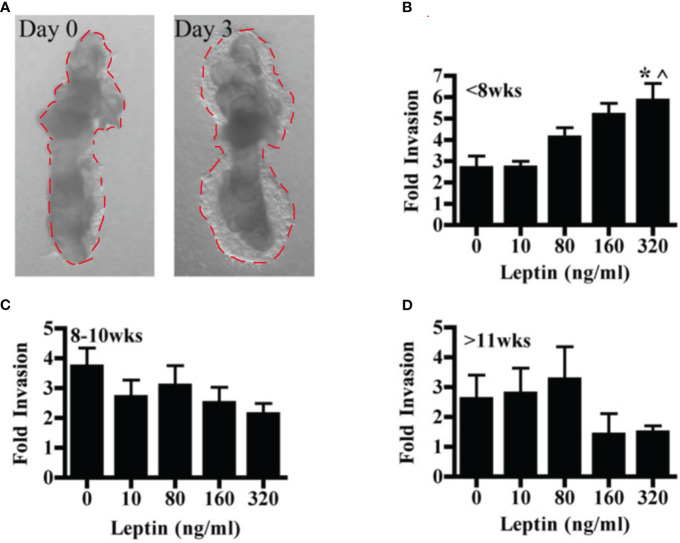
Leptin affects human explant CTB invasion in a dose and gestational age-dependent manner. Invasion of placental villous explants plated on Matrigel was evaluated in the presence of 0-320 ng/ml leptin. The area of Matrigel invaded by CTBs after three days of leptin treatment (Day 3) is normalized to the area invaded on Day 0, before the initiation of leptin treatment. Normalized data are combined from independent experiments. **(A)** A representative image of the CTB invasion on Days 0 and 3. **(B)** Fold change invasion of <8 weeks gestation explant CTBs increases with increasing leptin dose (5- and 7-week gestation placentas; n=6-8 explants per leptin concentration). **(C)** Fold change invasion of 8-10-week gestation explant CTBs is not differentially affected by leptin (8, 9, and 10-week gestation placentas; n=8-14 explants per leptin concentration). **(D)** Fold change invasion of explant CTBS from two independent 11-week gestations had a differentially invasive trend in response to leptin (n=5-6 explants per leptin concentration). Data are reported as mean fold change in the area invaded on Day 3 compared to Day 0 +/- SE. *p<0.05 vs. 0 ng/ml, ^p<0.05 vs. 10 ng/ml.

The effect of leptin on the invasion of CTBs in human placental villous explants varied with both dose and gestational age. Villous explants provide a model system for exploring the effects of leptin on CTB invasion in a context that maintains complex cellular interactions as well as the secretion of stromal and syncytial factors CTB invasion in villous explants was observed from Day 1 to Day 3. (**Figure 2A**). In the earliest samples, less than 8 weeks gestation, leptin promoted invasion with increasing dose, reaching significance at 320 ng/ml (**Figure 2B**). Leptin did not affect the invasion of explant CTBs from 8-10 weeks gestation (**Figure 2C**). However, leptin had a differentially invasive response in explant CTBs after 11 weeks of gestation, similar to what was observed for HTR-8/SVneo cells and pCTBs (**Figure 2D**).

### 3.3 Leptin does not affect MMP-2 and -9 activity in HTR-8/SVneo and pCTB

**Figure 3:**
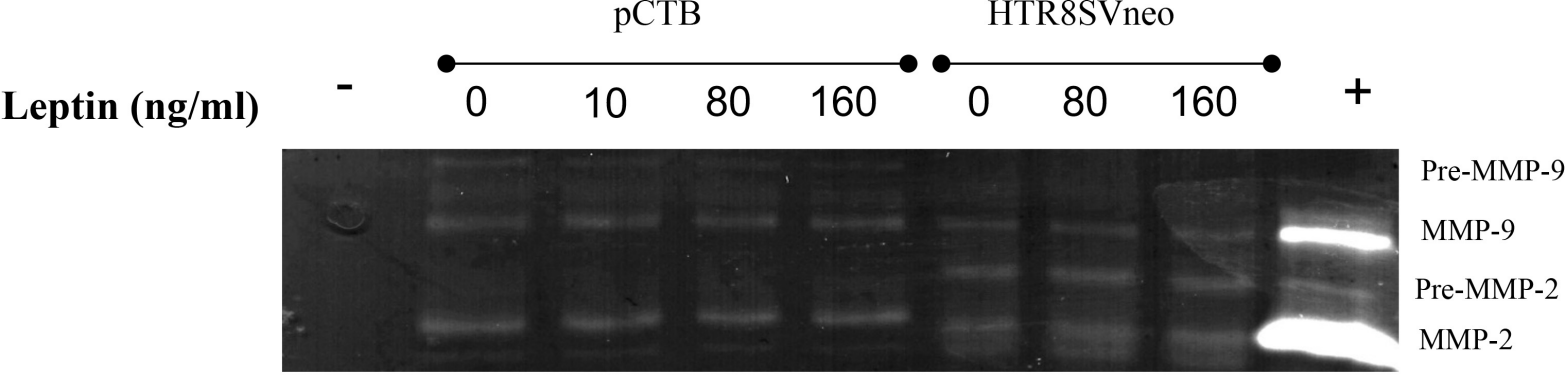
Leptin did not change MMP-2 or MMP-9 activity in human CTBs cultured on Matrigel. Culture medium from the top chamber of the invasion assays was collected and analyzed for MMP-2 and -9 activity by gelatin zymography. Representative images are shown from the conditioned medium after 24 hours for 17-week pCTBs and HTR-8/Svneo cells (conditioned medium from n=3 invasion assays). Similar results were seen for pCTB regardless of gestational age (n=4, 17-23 weeks gestation). Serum-free medium served as the negative control (-), and medium containing serum served as the positive control (+).

Leptin has been shown to alter MMP activity in murine as well as in human CTBs and may mediate the effect of leptin on invasion. Therefore, we evaluated the activity of MMP-2 and MMP-9 in the HTR-8/SVneo and pCTBs by gelatin zymography. HTR-8/SVneo cells expressed very low levels of active MMP-9 and differences in activity in response to leptin were not observed. HTR-8/SVneo cells also expressed pro- and active MMP-2, both were unaffected by leptin. Primary CTBs mainly expressed active MMP-9, and had very low levels of pro-MMP-2 and undetectable active MMP-2. MMP levels and activity were not affected by leptin concentration at any gestational age in pCTBs (**Figure 3**). The original gel image illustrating the enzymatic activity of MMP2 and MMP9 following leptin treatment in pCTB (17 weeks) and HTR8/SVneo cells is shown in **Supplemental Figure I**. As the villous explants are cultured in serum, which contains substantial MMP activity, we were not able to evaluate leptin effects on MMP activity in the explant system. Leptin effects on MMP-2 and -9 do not explain the differential effect of leptin on CTB invasion.

### 3.4 Leptin receptor expression does not change during the first half of pregnancy

**Figure 4:**
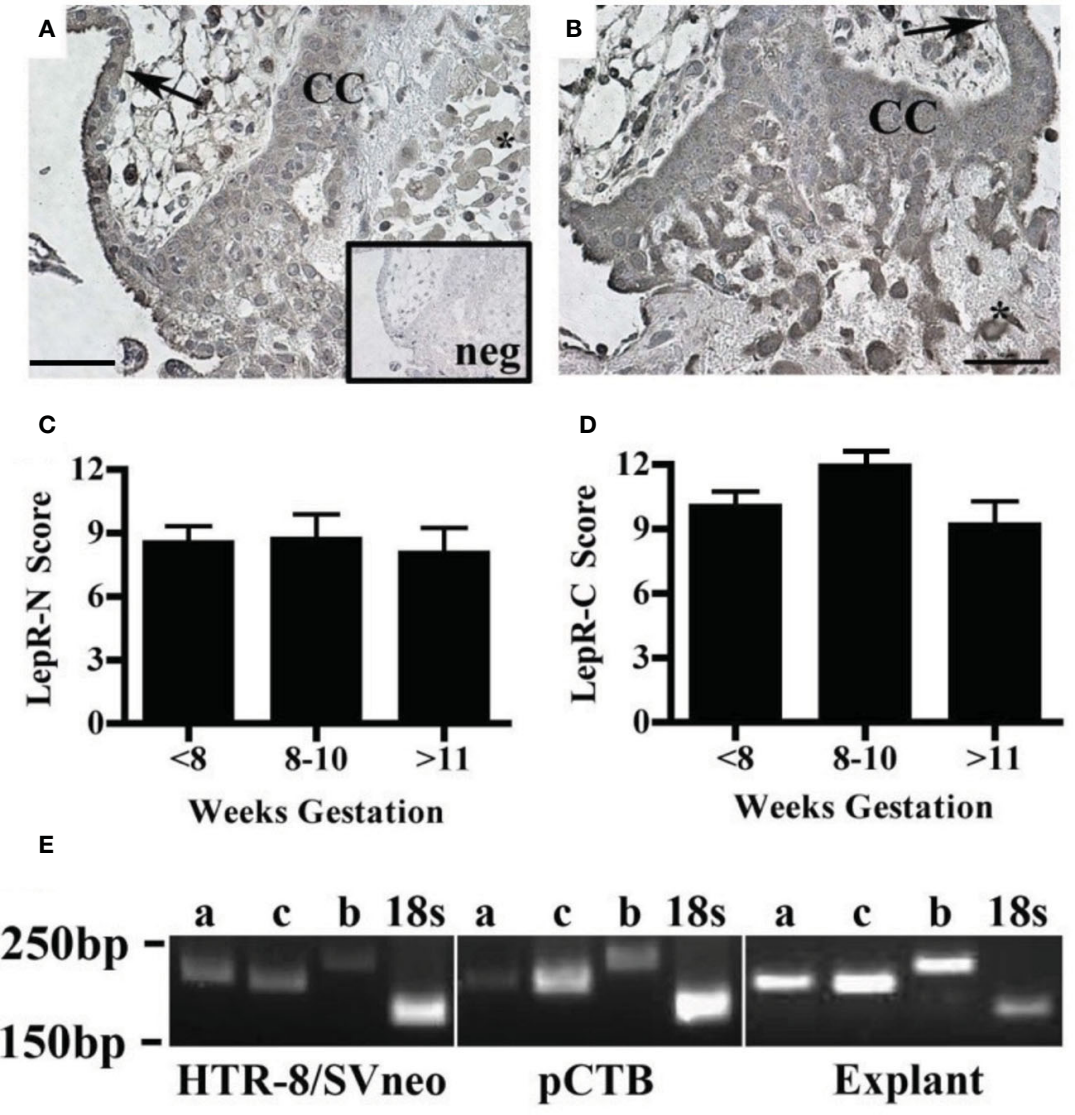
Human placental LepR expression did not change across the first half of gestation. Formalin-fixed, paraffin-embedded human placental biopsy tissue sections were evaluated by immunohistochemistry for LepR with antibodies that detect **(A)** all isoforms (N-20 antibody) or **(B)** exclusively the long isoform (C-20 antibody). LepR expression was found in all trophoblast populations at all gestational ages (<8 weeks n=8, 8-10 weeks n=6, 11-20 weeks n=7). Representative images containing villous and basal plate trophoblasts are shown; arrow indicates villous CTBS, CC indicates cell column CTBS, *indicates extravillous trophoblasts, and scale bar represents 50μm. The inset in A shows negative control. **(C)** Immunohistochemistry scores of LepR-N expressions (all isoforms) and **(D)** LepR-C expression (long isoform only). An intensity score (0-3) was multiplied by the percent-positive trophoblasts score (<10%=1, 10-50%=2, 50-90% =3, >90%=4) for each sample evaluated at 10X magnification. **(E)** RT-PCR was performed using LepR isoform-specific primers [a=short isoform 2 (212bp), c=short isoform 3 (204bp), b-long isoform 1 (244bp)] on total mRNA from all three model systems; 18S (186bp) was used as an amplification control. For all model systems and all gestational age windows evaluated all three isoforms were expressed. Gel blots are representative of HTR-8/Svneo (n=3), pCTB (n=6; 24 weeks shown), and explants (n=4; 11 weeks shown).

The effect of leptin on CTB invasion could be mediated by LepR. Therefore, we characterized leptin receptor expression in our model systems. Immunohistochemistry was used to localize the receptors by cell type and across gestation up to 20 weeks. We analyzed the villous explants used in the invasion assays as well as biopsies from additional placentas. Two different LepR antibodies were used to isolate isoform-specific expression. The first antibody recognizes an N-terminal antigen and detects all LepR isoforms (N-20). This antibody showed immunoreactivity in all trophoblasts at all gestational ages including syncytiotrophoblasts and CTB in the villi, CTBs in the cell column, and extravillous trophoblasts (**Figure 4A**). The second antibody recognizes a C-terminal antigen unique to the long receptor isoform (C-20). Immunoreactivity with this antibody was observed in syncytiotrophoblasts and CTB in the villi, CTBs in the cell column, and extravillous trophoblasts (**Figure 4B**). A comparison of LepR expression by semi-quantitative scoring of immunoreactivity did not reveal differences in expression from 5-20 weeks (**Figures 4C, D**). Because the long LepR isoform is expressed in all trophoblast populations at all gestational ages evaluated, we are unable to make conclusions about the short LepR isoforms from immunohistochemistry studies. We, therefore, conducted RT-PCR using isoform-specific primers to determine if the short isoforms (LepRa and LepRc) as well as the long isoform (LepRb) is expressed in our model systems. In HTR-8/SVneo cells, pCTBs, and explant tissue, mRNA for all three isoforms were expressed (**Figure 4E**).

### 3.5 Leptin receptor expression does not change with differential leptin treatment in HTR8/SVneo cells

**Figure 5:**
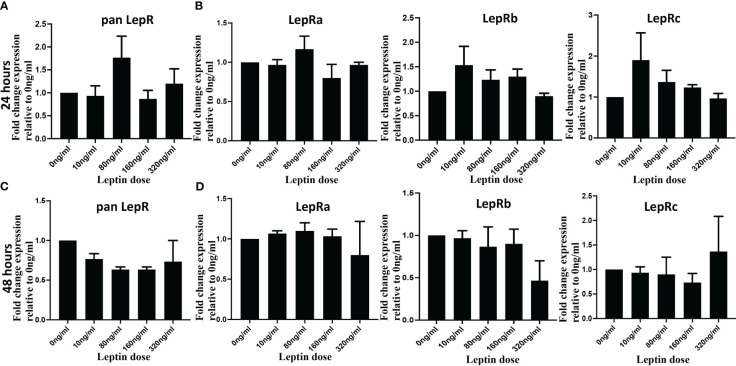
Leptin did not change LepR expression in HTR8/SVneo cells. HTR8/SVneo cells were treated with indicated concentration of leptin and total RNA was isolated 24 hours (Upper panel) and 48 hours (Lower panel) hours post-treatment. Total RNA was used to perform qPCR to determine mRNA levels of Leptin receptor using pan Lep R primers **(A, B)** and isoform specific primers **(C, D)**. Bar graphs represent the mean fold change relative to the control (Ong/ml). The gene expression values for LepR at each dose were normalized to 18S. Data are depicted as Mean ± SEM from three independent experiments.

Leptin’s impact on the invasion of cytotrophoblasts (CTB) may be attributed to potential variations in LepR expression in response to varying concentrations of leptin. To investigate whether an increasing dose of leptin induces or downregulates leptin receptor expression, HTR8/Svneo cells were treated with 0, 10, 80, 160, and 320 ng/ml of leptin for 24 and 48 hours. Quantitative polymerase chain reaction (qPCR) analyses were performed using both pan LepR and isoform-specific primers to assess the mRNA expression levels of distinct LepR isoforms. The results revealed no statistically significant differences in LepR expression in HTR8/Svneo in response to increasing doses of leptin. Although at 24 hours, panLepR expression shows a bell-shaped response to increasing concentration of leptin, the differences were statistically non-significant (**Figure 5A**). Next, using HTR8/SVneo cells we explored if any of the Lep R isoform expression is impacted by increasing leptin dose and could be attributed differentially invasive phenotype. Further, LepR isoform-specific expression also did not show any significant change in response to varied leptin doses (**Figure 5B**). Similar results were obtained at 48 hours (**Figures 5C, D**). Of interest, even the fold change ratios of long to short isoforms did not show any significant changes in response to leptin doses (**Supplementary Figure II**). This indicates a lack of modulation in leptin receptor expression under the experimental conditions. Due to the limited availability of tissue samples and primary CTB cell numbers, we were unable to replicate the same assay in the other two model systems (pCTBs and villous explants). While the ratios of long/short LepR isoforms did not change in response to leptin, however, we cannot rule out the possibility that the relative ratio of short LepR isoforms can change over gestation.

### 3.6 Leptin has a dose-dependent effect on signaling pathways

Next, we were interested in unraveling the molecular mechanism of leptin’s differential effect on human trophoblast invasion. One possibility for the differential invasion in normal trophoblasts in response to different concentrations of leptin is the differential activation of cell signaling pathways. An untargeted approach was implemented to evaluate 46 unique protein phosphorylations in response to 0, 80, and 320 ng/ml of leptin in HTR-8/SVneo cells. The phosphorylation signal on the arrays in response to leptin was normalized to signal in the absence of leptin (represented by the y-axis set to 1). A total of 16 proteins showed greater than a 20% difference in phosphorylation between 0, 80, and 320ng/ml leptin. Three different patterns of phosphorylation emerged. The proteins showing the same level of phosphorylation at 80 and 320 ng/ml of leptin were categorized as “uninvolved”, and those showing increased phosphorylation at 80ng/ml of leptin and less phosphorylation at 320ng/ml of leptin were designated as “Bell-shaped”. The proteins that showed increased phosphorylation at 80ng/ml of leptin and a further increase in phosphorylation at 320ng/ml of leptin were categorized as “further augmented”. Each pattern was given a letter designation (U= uninvolved, B= bell-shaped, F= further augmentation). Less phosphorylation or increased phosphorylation at 320ng/ml of leptin could result in reduced invasion. The proteins were grouped by signal transducer and activator of transcription proteins (STATs), phosphoinositide-3-kinase (PI3K), and Mitogen-activated protein kinase (MAPK) pathways (pathways typically associated with leptin signaling).

**Figure 6:**
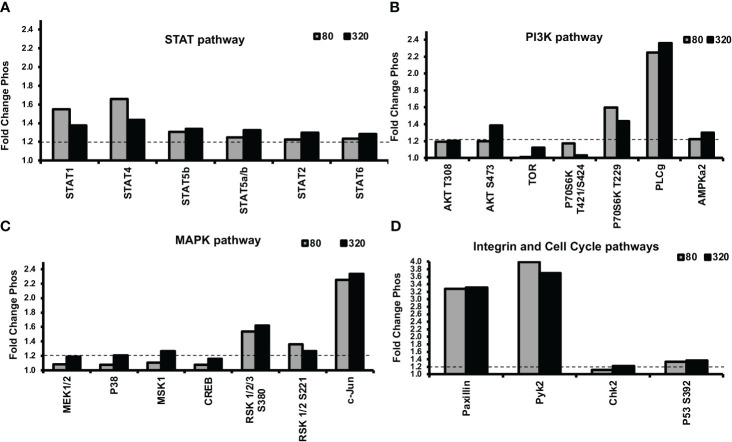
Alterations in STAT, PI3K, and MAPK signaling protein phosphorylation were evident in response to leptin, as determined by a phosphokinase array. HTR-8/SVneo cells, cultured on matrigel for an hour were treated with 0, 80, or 320ng/ml leptin for 30 minutes and were subjected to phosphokinase array analysis. Out of 46 unique phosphorylation sites evaluated, 16 sites showed greater than a 1.2-fold (20%) difference in phosphorylation between 0, 80, and 320ng/ml leptin. The dotted line represents a threshold of 1.2- fold. Leptin doses 80 and 320ng/ml are shown as gray and black bars respectively. Changes in phosphorylation in **(A)** STAT pathway, **(B)** PI3K pathway **(C)** MAPK pathway, and **(D)** Integrin and Cell Cycle Pathways. STAT, PI3K, and MAPK signaling may be involved in the differential invasive response to leptin.

Many of the STATs showed increased phosphorylation in response to leptin treatment (**Figure 6A**). STATs 1 and 4 showed bell-shaped pattern phosphorylation and may be involved in the differential invasion of trophoblasts in response to leptin. The remaining STATs did not show patterns suggestive of involvement in the bell-shaped pattern. STATs 5, 2, and 6 showed pattern U phosphorylation; STAT3 was not found to be induced by 30 minutes of leptin treatment.

The PI3K pathway also showed a phosphorylation response to leptin treatment (**Figure 6B**). AKT, a member of the PI3K pathway, showed site-specific phosphorylation at different leptin doses. Site T308, which PDK-1 phosphorylates, was equally stimulated by both doses (80 and 320ng/ml) of leptin, suggesting a U pattern whereas site S473, which is phosphorylated by mTORC2, suggests an F pattern of phosphorylation. mTOR showed increased phosphorylation at 320 ng/ml whereas 80ng/ml was no different from baseline, suggesting an F pattern of phosphorylation. P70 S6 kinase, which is downstream of mTOR’s isoform, mTORC1, showed no phosphorylation at site T389, on the contrary, its site T229 (phosphorylated by PDK1) and T421/S424 (phosphorylated by MAPK) showed B pattern phosphorylation. These observations suggest that the differential leptin invasive response may be driven through AKT/mTOR/p70 S6.

Many proteins in the MAPK pathway were phosphorylated in response to leptin treatment (**Figure 6C**). Two transcription factors c-jun and Adenosine 3’5’ cyclic monophosphate (cAMP) response element binding protein (CREB) showed F pattern phosphorylation. MKK1/2(MEK1/2), p38, and mitogen and stress-activated protein kinase (MSK-1) also showed F pattern phosphorylation where only 320ng/ml leptin resulted in greater than a 20% increase in phosphorylation compared to no leptin. P42/p44 phosphorylation was undetected at 30 mins of leptin stimulation. P90 ribosomal S6 kinase (RSK)-1/2/3, showed U pattern phosphorylation at both S380 and S221 activation sites, which does not support the role of RSK in the differential invasion. The phosphorylation array data suggest that many proteins in the MAPK stress response pathways are stimulated by 320ng/ml leptin, including transcription factors which may ultimately be inhibiting invasion at 320ng/ml leptin.

Other proteins that were phosphorylated in response to leptin include the cell cycle pathway proteins (Chk2 and p53) and integrin pathway proteins [Paxillin and non-receptor proline-rich tyrosine kinase (pyk-2)] (**Figure 6D**). Chk2 and p53 showed U pattern phosphorylation and hence are unlikely to be involved in differential invasive response to leptin. Pyk-2 showed B-pattern phosphorylation however the other members of the integrin signaling complex such as Paxillin, FAK (Focal adhesion kinase), and Src in contrast showed no change in phosphorylation with leptin treatment (data not shown). However, we speculate that leptin and integrin signaling could be involved in crosstalk as evidenced by differences in Pyk-2 phosphorylation at 80 and 320ng/ml of leptin dose.

## 4 Discussion

This study shows that the effects of leptin on human CTB invasion are gestational age and dose-dependent. Our hypothesis that high levels of leptin would inhibit trophoblast invasion did not fully capture the complexity of human CTB responses to leptin over gestation. Before 8 weeks gestation, leptin promoted explant CTB invasion in a positive dose-response manner. Between 8-10 weeks gestation, leptin had no significant effects on explant CTB invasion. After 11 weeks gestation, leptin demonstrate a differential invasive response in explant CTBs, HTR-8/SVneo cells, and pCTBs model systems revealing a complex relationship between leptin and invasion over gestation. We investigated leptin’s impact on MMP activity in our model systems (HTR8/SVneo cells and pCTB) and found that MMP levels and activity were not affected by leptin concentrations. The effect of leptin on CTB invasion could be mediated by LepR and hence we confirmed that all the isoforms of LepR are expressed in our model systems. We also determined that LepR isoform expression does not change in HTR8/Svneo cells when exposed to differential leptin dose. This led us to explore if differential leptin concentrations might activate distinct signaling pathways, influencing CTB invasion. Unbiased exploration of signaling in response to leptin concentrations in HTR-8/SVneo cells suggests roles for several STAT, PI3K, and MAPK signaling pathway molecules in the differential invasive response to leptin.

In early gestation, human CTBs are highly invasive as they establish the maternal/fetal interface. In the second trimester, the invasive capacity decreases and is complete by the end of the second trimester. Thus, it is conceivable that CTB’s ability to respond to invasive stimuli (i.e., leptin) would change with gestation. Our results could reflect that leptin in early gestation signals CTBs to invade. In mid-gestation, moderate levels of leptin continue to enhance invasion. Gestational age-dependent responses have also been found for murine CTBs, where leptin promoted the invasion of primary CTBs isolated at day 10 of gestation but had no effect on murine CTBs isolated from day 18 (mice have a 21-day gestation) (13). Although both human CTBs and murine CTBs show changes in responsiveness to leptin over gestation, the character of these responses is different. Our data shows a bell-shaped invasive response to leptin that is distinct from what has been observed in murine models. However, there is a report of leptin dose-response in HTR8/SVneo cells consistent with findings in murine models (43). It is noteworthy, that we were able to demonstrate the bell-shaped invasive response in primary CTBs which corroborates with our findings in HTR8/SVneo cells conferring confidence in the reproducibility and relevance of our data.

The leptin effects on invasion were not explained by MMP-2 or -9 activity. Leptin was previously found to stimulate MMP-2 and/or -9 activity in primary human CTBs isolated from early, but not late, gestation when cultured on glass or plastic; mid-gestation was not evaluated in those studies (14, 44). Differences between our results and previous studies may be due to differences in culture conditions. Culturing primary human CTBs on glass/plastic induces them to differentiate toward a syncytial phenotype, whereas culture on Matrigel induces differentiation toward an invasive phenotype. These different cell types very well may have different responses to leptin. Further, MMP activity may be maximal in CTBs differentiated on Matrigel and therefore not able to be further stimulated by leptin. Our results suggest that some other aspect of invasion rather than MMP-2 or -9 activity is responsible for the differential response to leptin.

The question remains why does the CTB response to leptin change over gestation? The cells may become leptin-resistant with increasing gestational age. However, our data do not support this idea as human CTBs isolated throughout the second trimester retain the ability to increase invasion in response to 80 ng/ml leptin. An alternative possibility is that human CTBs are engaging different signaling machinery, potentially through the expression of different receptors. Regarding LepR, our data indicate that all LepR isoforms are present at all gestational ages and in all our model systems. These findings are consistent with those of other groups (6, 45, 46). Changes in either total LepR or long/short LepR isoform levels over gestation or in response to leptin dose itself do not appear to account for the change in responsiveness to leptin.

A differential response of cells to leptin is not without precedent. Human monocytes increase cholesterol synthesis in response to leptin up to 100 ng/ml but suppress cholesterol synthesis at higher concentrations. At lower concentrations, monocytes engage mitogen-activated protein kinase (MAPK) and phosphatidyl-3-kinase (PI3K) signaling, but actions at higher concentrations involve protein kinase C (PKC) signaling (47). Subsets of rat ventromedial and accurate hypothalamic nuclei neurons have a differential excitation/inhibition response to leptin, also possibly mediated by PI3K and AMP-activated protein kinase (AMPK) (48). The differential leptin action on human CTB may similarly be regulated by engaging different signaling pathways at different concentrations. LepR signaling is also known to be subject to negative feedback by suppressor of cytokine signaling (SOCS)-3. SOCS-3 levels or other signaling pathway molecules may change over gestation or in response to leptin dose. Another possible mechanism is leptin impacting the degree of LepR extracellular cleavage, thereby altering the ability of LepR to signal. Increasing doses of leptin up to 100ng/mL has been shown to decrease LepR cleavage and therefore increase cellular responsiveness to leptin (49). This maybe contributing to the increase in invasion response in our data from 0 to 80ng/mL treatments. Whether higher doses of leptin would further decrease LepR cleavage not known, but if holds then would not explain the decreasing response to leptin at the higher doses. Another contributing factor to the differential response of cells to leptin could be saturation of LepR which has been previously reported in pathological pregnancies (50). It has been observed in trophoblast explants that increasing dose of leptin can increase signaling through LepR (51) but overstimulation of LepR leads to negative modulation of signaling pathways (52). Alternatively, LepR signaling capacity may change via alterations in membrane localization. In other tissues, high leptin levels induce receptor internalization which (53, 54), if occurring in our systems, may result in a loss of response to leptin at higher concentrations.

To determine which signaling pathways are involved in the leptin-induced differential invasive response of trophoblast cells, we implemented an untargeted approach using a phosphokinase array on the HTR-8/SVneo cell model system. The phosphorylation data suggest the roles of STAT, PI3K, and MAPK signaling in the differential invasive leptin response in HTR8/SVneo cells. STAT1 and STAT4 showed a differential pattern of phosphorylation. STAT1 is known to be phosphorylated by leptin signaling through LepR. Further, STAT1 expression is known to be increased in the processes of CTB differentiation into EVTs. The expression of STAT1 in EVT suggests it may be involved in the process of trophoblast invasion. STAT4 has not previously been reported to be phosphorylated by leptin signaling through LepR. STAT4 is generally thought of as being involved in immune responses. However, as with STAT1, STAT4 expression is increased in the process of CTB differentiation to EVT. The promoters of many genes upregulated in EVT have putative STAT4 response elements further adding support to the role of STAT4 in trophoblast invasion (55). The differential phosphorylation pattern of STAT1 and STAT4 responses to leptin as well as their association with trophoblast invasion and differentiation suggest these may be involved in the differential response and warrant follow-up studies.

Many proteins in the MAPK pathway were phosphorylated in response to leptin treatment. Two transcription factors, c-jun and CREB showed F pattern phosphorylation. C-jun is involved in diverse processes in many different tissues. CREB also affects many processes including cell migration and differentiation. MKK1/2(MEK1/2), p38 and MSK-1 also showed F pattern phosphorylation. p38 can activate MSK-1 which can in turn activate CREB. MAPK pathway data suggested that many proteins in the MAPK stress-responsive pathways are stimulated by 320ng/ml leptin, including transcription factors, which may ultimately contribute reduction in the invasion at 320 ng/ml.

PI3K/AKT signaling pathway may also play a role in leptin-induced CTB invasion, but it is not likely in response to PI3K activation. Rather, leptin may be signaling through the AKT/mTOR/p70 S6 kinase pathway to alter invasion.

Specifically, STAT1/4, the p38 stress pathway, and p70 S6 kinase proteins look promising for involvement in the failure of invasion at high leptin doses. mTOR/p70 S6 kinase inhibition has been shown to inhibit cell proliferation and invasion in trophoblast cell models (56). The phosphorylation data suggest that STAT, PI3K, and MAPK signaling may be involved in the differential invasive response of leptin in HTR-8/SVneo cells and warrants further investigation. Although the invasion pattern of pCTBs and HTR-8/SVneo cells show similar response to leptin, the signaling cascades in HTR-8/SVneo cells maybe different, given it is a transformed cell line, than those in pCTBs derived from primary tissue. Nonetheless, the HTR-8/SVneo results allude to the pathways that can be involved in the differential invasive response of leptin. Inhibiting and activating these pathways in the various model systems would further clarify how these pathways are involved in the loss of leptin-induced invasion at the higher doses.

When we consider hyperleptinemia observed in preeclampsia, a disorder of insufficient trophoblast invasion, excess leptin may be hindering placental invasion as part of the pathologic process. While all women with obesity do not develop PE, our data provides a potential mechanism explaining the association of obesity with PE. Women who enter pregnancy with higher levels of leptin may have a leptin response in the second trimester that does not optimally promote invasion. There are several possibilities for how excess leptin could prevent maximal CTB invasion in PE. Our data support that exposure of CTBs to higher levels of leptin after 11 weeks could create a situation where the trophoblasts fail to invade to a physiologic depth. Our studies would suggest that the effect of leptin on CTB invasion in PE may be a second-trimester-specific defect. This concept is consistent with the finding that women who have higher leptin levels at 13 weeks gestation are at higher risk of subsequently developing PE (31, 32). Another possibility, which our studies do not address, is that CTBs from placentas of pregnancies complicated by PE may have an inherent defect, which prevents them from responding to the leptin-induced promotion of invasion.

## 5 Conclusion

This work looks at the direct effect of leptin on first and second-trimester human CTB invasion. Leptin has varying yet reproducible effects dependent on leptin dose and the gestational age of the tissue tested. Leptin differentially modulates human CTB invasion without affecting MMP-2 and MMP-9 activities or LepR isoform expression. While changes in leptin responsiveness are not due to differences in LepR expression our data suggest that they may be due to differential downstream signaling. This work demonstrates that the HTR-8/SVneo cell models in regards to leptin response recapitulates second trimester pCTBS and therefore can be used for further understanding of the differential response of human CTBs to leptin. Further, this work provides fundamental information for considering potential mechanisms of how elevated maternal leptin levels may contribute to the pathogenesis of PE, through alterations in CTB invasion. Clarifying the role of LepR and leptin signaling in the differential invasive response of primary trophoblasts and confirming this response in recently developed trophoblast stem cell models (57) will provide important insights into the molecular mechanisms involved in regulators of CTB invasion and the potential role in the pathogenesis of PE.

## Data availability statement

The raw data supporting the conclusions of this article will be made available by the authors, without undue reservation.

## Ethics statement

The studies involving humans were approved by University of Colorado Institutional Review Board. The studies were conducted in accordance with the local legislation and institutional requirements. The ethics committee/institutional review board waived the requirement of written informed consent for participation from the participants or the participants’ legal guardians/next of kin because human samples were obtained from discarded tissue provided by the clinic. Patients signed a surgical consent form that provided an opt-in/opt-out option for procedure tissues to be used for research. There was no direct consent required for this study as approved by the local IRB.

## Author contributions

KR: Conceptualization, Writing – review & editing, Data curation, Formal Analysis, Investigation, Methodology, Software, Validation, Visualization, Writing – original draft. SS: Data curation, Formal Analysis, Investigation, Methodology, Software, Validation, Visualization, Writing – original draft, Writing – review & editing. KB: Writing – original draft, Methodology. AK: Methodology, Writing – original draft. VW: Conceptualization, Funding acquisition, Project administration, Supervision, Writing – review & editing.
