# Supplementary figures and images for: The effects of leptin on human cytotrophoblast invasion are gestational age and dose-dependent
Source: Front Endocrinol (Lausanne). 2024 May 23;15:1386309. doi: 10.3389/fendo.2024.1386309 (PMC11154010; doi:10.3389/fendo.2024.1386309)

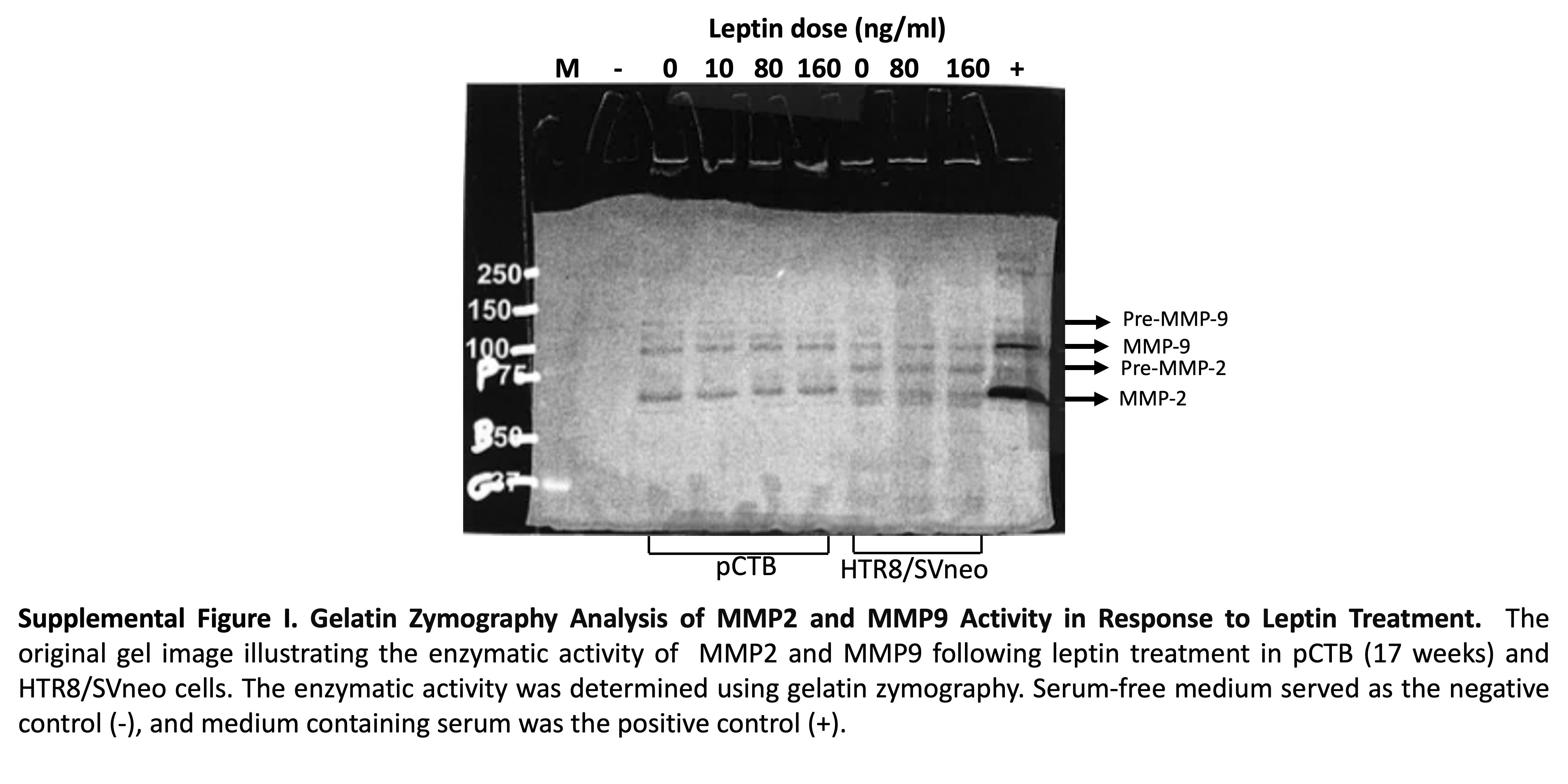

Supplement: Supplementary file 1 [file Image_1.jpg]

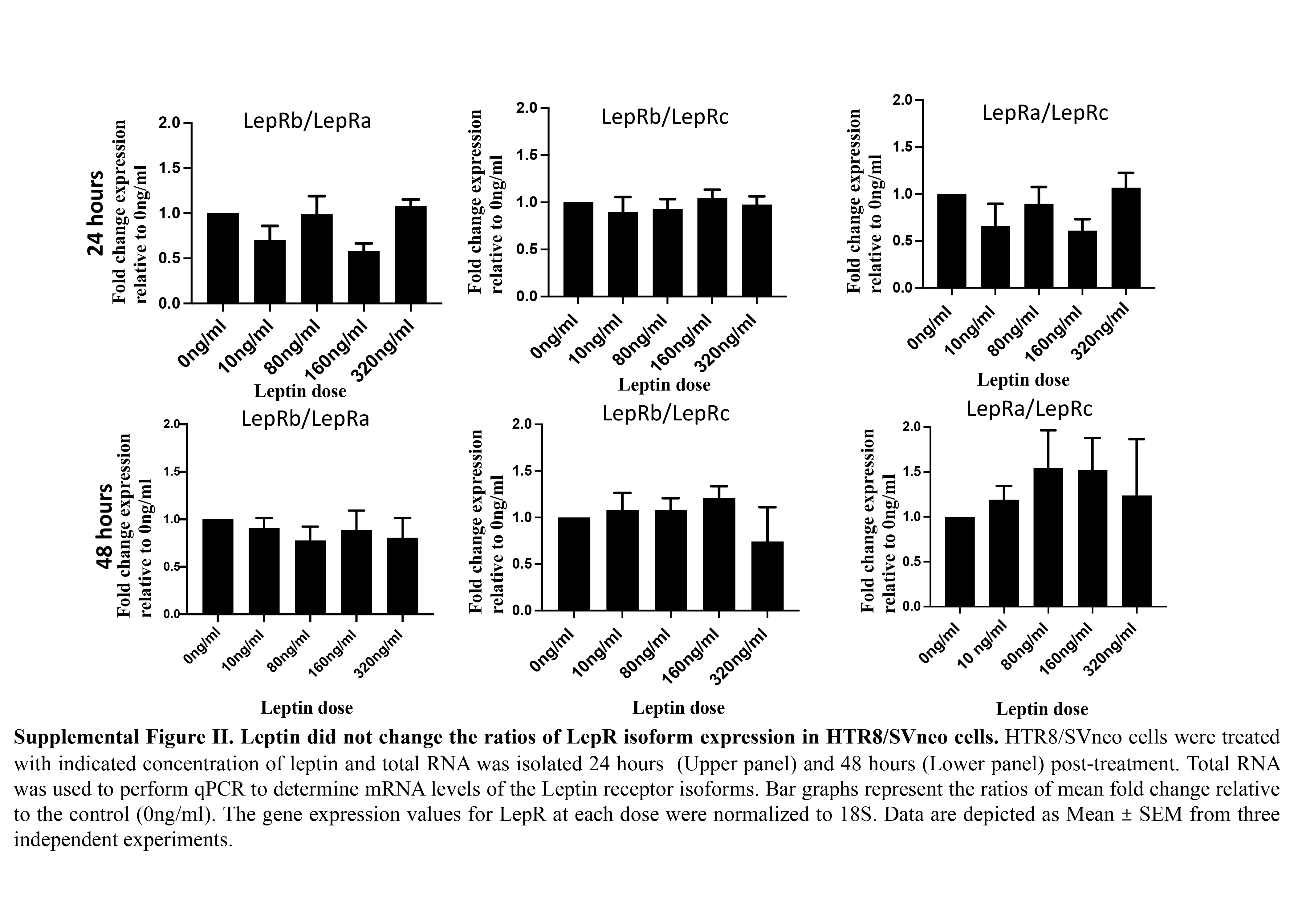

Supplement: Supplementary file 2 [file Image_2.jpeg]
